# Do Perineural Dexamethasone and Dexmedetomidine Improve Analgesia and Inflammatory Response After Lumbar Spine Surgery?

**DOI:** 10.1097/CORR.0000000000004008

**Published:** 2026-06-05

**Authors:** Tomasz Reysner, Bahadir Ciftci, Selcuk Alver, Katarzyna Wieczorowska-Tobis, Agnieszka Neumann-Podczaska, Małgorzata Reysner

**Affiliations:** 1 Department of Anesthesiology and Intensive Therapy, Pathophysiology of Pain Unit, Poznań University of Medical Sciences, Poznań, Poland; 2 Department of Anatomy, Istanbul Medipol University, Istanbul, Turkey; 3 Department of Anesthesiology and Reanimation, Istanbul Medipol University, Istanbul, Turkey; 4 Department of Anesthesiology and Reanimation, Biruni University Hospital, Istanbul, Turkey; 5 Department of Palliative Medicine, Poznań University of Medical Sciences, Poznań, Poland; 6 Department of Medical and Health Sciences, Senior Institute, Vizja University, Warsaw, Poland; 7 Department of Clinical Anesthesiology and Pain Management, Poznań University of Medical Sciences, Poznań, Poland

## Abstract

**Background:**

Postoperative pain after lumbar spine surgery often requires substantial opioid use. Perineural adjuvants such as dexamethasone and dexmedetomidine are increasingly used to prolong the duration of regional blocks and improve analgesic quality; however, their comparative effects remain uncertain. Ultrasound-guided erector spinae plane block (ESPB) is widely used as part of multimodal analgesia, but the selection of perineural adjuvants remains uncertain.

**Questions/purposes:**

(1) Does adding adjuvants (dexamethasone or dexmedetomidine) to the ESPB result in longer time to first rescue opioid? (2) Do patients who received adjuvants have lower total opioid consumption in the first 48 hours after surgery, and do they have lower pain scores? (3) Does dexamethasone or dexmedetomidine result in lower postoperative systemic inflammatory indices?

**Methods:**

This study was conducted at a tertiary academic referral center specializing in spine surgery. In this prospective, randomized, triple-blinded controlled trial, between March 2024 and December 2025, a total of 103 patients were assessed for eligibility. Of these, 94 were randomized. Four patients were excluded after randomization because of intraoperative changes in surgical plan or hemodynamic instability, leaving 96% (90) of adult patients undergoing posterior lumbar decompression with instrumented fusion. These patients were randomly assigned to receive ESPB in three groups: the ropivacaine alone (control), the ropivacaine plus dexamethasone, and the ropivacaine plus dexmedetomidine groups. The primary outcome was time to first rescue opioid administration. Secondary outcomes included cumulative opioid consumption within 48 hours, postoperative pain scores, and systemic inflammatory markers (neutrophil-to-lymphocyte ratio, platelet-to-lymphocyte ratio, and systemic immune-inflammation index). Patients were followed for 48 hours postoperatively.

**Results:**

Patients receiving dexamethasone or dexmedetomidine required rescue opioids later than those receiving ropivacaine alone. The mean ± SD time to first opioid administration was 6.2 ± 0.8 hours with ropivacaine alone versus 12.7 ± 2.7 hours with dexamethasone (mean difference 6.6 hours [95% confidence interval (CI) 5.6 to 7.7]; p < 0.001) and 15.5 ± 2.0 hours with dexmedetomidine (mean difference 9.6 hours [95% CI 8.5 to 10.5]; p < 0.001). Postoperative pain scores were reduced in both adjuvant groups at all assessed time points. Compared with dexmedetomidine and ropivacaine alone, dexamethasone was associated with lower postoperative systemic inflammatory indices, indicating greater attenuation of the inflammatory response.

**Conclusion:**

Dexamethasone and dexmedetomidine demonstrate distinct and complementary effects when used as adjuvants to ESPB in lumbar spine surgery. Dexmedetomidine primarily enhances early postoperative analgesia, whereas dexamethasone more effectively reduces cumulative opioid consumption and systemic inflammation. These findings support a clinically actionable, mechanism-informed approach to selecting ESPB adjuvants according to the desired temporal analgesic profile and risk of prolonged postoperative opioid requirement.

**Level of Evidence:**

Level I, therapeutic study.

## Introduction

Postoperative pain after lumbar spine surgery remains a major clinical challenge, often necessitating substantial opioid administration and contributing to delayed recovery, opioid-related adverse effects, and prolonged hospitalization [6]. Pathways for enhanced recovery after surgery emphasize multimodal, opioid-sparing analgesia, with regional anesthesia techniques playing an increasingly central role [9, 18, 19].

The erector spinae plane block (ESPB) has emerged as a promising regional analgesic technique for spine surgery [23]. By depositing local anesthetic in the fascial plane deep to the erector spinae muscle, ESPB allows for multilevel craniocaudal spread and indirect modulation of dorsal rami and paravertebral structures [31]. Clinical studies have demonstrated that ESPB reduces postoperative pain scores and opioid consumption in lumbar and thoracic spine procedures [6, 23, 31]. However, variability in block duration and the occurrence of rebound pain remain important limitations.

Perineural adjuvants such as dexamethasone and dexmedetomidine have been widely used to prolong the duration and enhance the quality of peripheral nerve blocks [4, 20]. Dexamethasone exerts potent antiinflammatory effects and has been shown to prolong block duration through both local and systemic mechanisms [15, 22]. Dexmedetomidine, an α2-adrenergic agonist, may enhance analgesia through both peripheral and central mechanisms; however, the relative contribution of local perineural versus systemic effects remains incompletely defined in fascial plane blocks such as ESPB [10]. Despite extensive use, the comparative effects of these agents on postoperative pain dynamics of systemic inflammatory response in spine surgery remain incompletely understood [24].

Importantly, postoperative pain after major spine surgery is not solely a nociceptive phenomenon but is closely intertwined with the surgical stress response and systemic inflammation [13]. Biomarkers such as the neutrophil-to-lymphocyte ratio, platelet-to-lymphocyte ratio, and the systemic immune-inflammation index (SII) have gained attention as integrative indicators of postoperative inflammatory burden and may provide insight into mechanisms underlying rebound pain and prolonged opioid requirement [12, 16, 29].

Inadequately controlled postoperative pain and exaggerated inflammatory response after spine surgery may delay mobilization, impair participation in rehabilitation, and negatively affect adherence to enhanced recovery pathways.

Therefore, in this study, we asked: (1) Does adding adjuvants (dexamethasone or dexmedetomidine) to the ESPB result in longer time to first rescue opioid? (2) Do patients who received adjuvants have lower total opioid consumption in the first 48 hours after surgery, and do they have lower pain scores? (3) Does dexamethasone or dexmedetomidine result in lower postoperative systemic inflammatory indices?

## Patients and Methods

### Study Design

This prospective, randomized, triple-blinded, parallel-group clinical trial was conducted at a tertiary academic referral center specializing in orthopaedic surgery. The first patient was enrolled on March 1, 2024.

### Participants

Between March 2024 and December 2025, adult patients scheduled for elective primary posterior lumbar decompression and multilevel spinal fusion with instrumentation were screened for eligibility. Inclusion criteria were patients who were 18 years or older, with an American Society of Anesthesiologists (ASA) physical status of I to III, and who were eligible for ESPB. Exclusion criteria included refusal to participate, history of opioid abuse, infection at the planned block site, ASA physical status IV to V, or contraindications to regional anesthesia. Although patients with opioid abuse were excluded, detailed quantification of prior intermittent opioid exposure for chronic degenerative spine pain was not prospectively collected. A total of 103 patients were assessed for eligibility; 94 were randomized and 87% (90 of 103; 30 per group) completed the study and were included in the final analysis (Fig. 1). Baseline demographic, clinical, surgical, pain, and inflammatory characteristics were comparable across groups (Table 1).

**Fig. 1:**
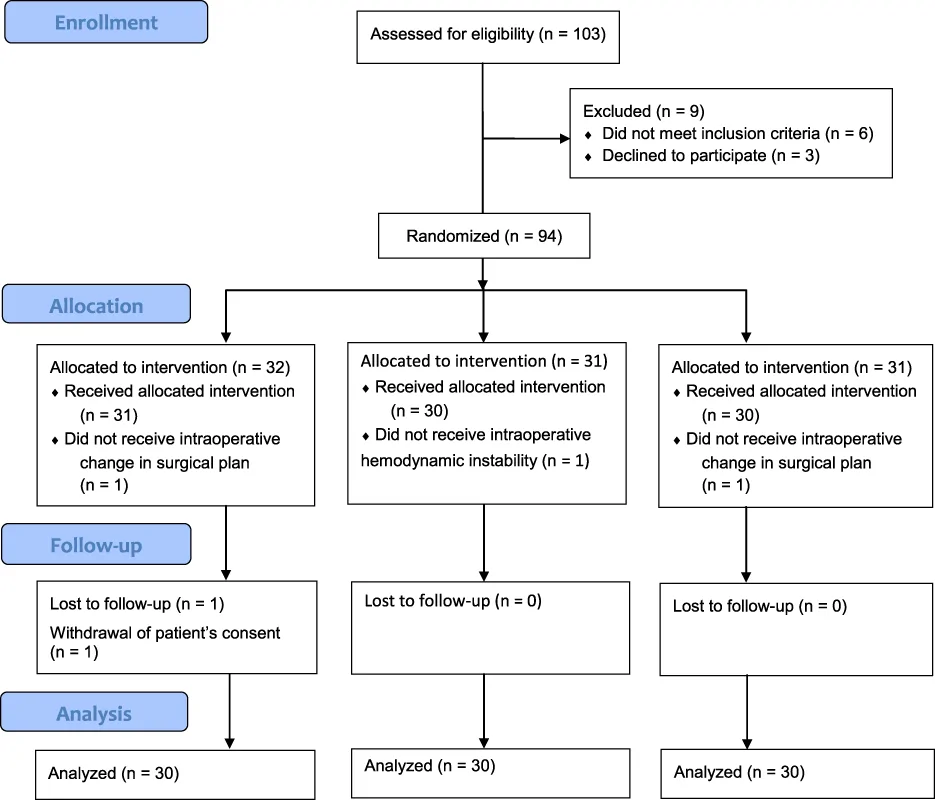
The CONSORT flow diagram illustrating patient enrollment, randomization, allocation, follow-up, and analysis in this RCT, which included 94 patients who were randomized and allocated to one of three study groups. Eighty-seven percent (90 of 103; 30 per group) of patients completed the study and were included in the final analysis.

**Table 1.** Baseline characteristics of the study sample

Variable	Ropivacaine alone (n = 30)	Ropivacaine with dexamethasone (n = 30)	Ropivacaine with dexmedetomidine (n = 30)
Age in years	63 ± 10	61.9 ± 10	62.7 ± 10
Gender, men^a^	47 (14)	50 (15)	43 (13)
BMI in kg/m^2^	27.8 ± 4.3	27.1 ± 4.0	28.0 ± 4.6
ASA physical status			
I	17 (5)	20 (6)	17 (5)
II	57 (17)	53 (16)	60 (18)
III	27 (8)	27 (8)	23 (7)
Diagnosis			
Degenerative lumbar stenosis	60 (18)	57 (17)	63 (19)
Spondylolisthesis	27 (8)	30 (9)	23 (7)
Other degenerative disease	13 (4)	13 (4)	13 (4)
Number of fused levels	2.6 ± 0.8	2.5 ± 0.7	2.7 ± 0.9
Duration of surgery in minutes	186 ± 42	179 ± 38	183 ± 40
Intraoperative blood loss in mL	320 ± 180	300 ± 170	330 ± 190
Baseline NRS score (preoperative)	6.8 ± 1.1	6.7 ± 1.0	6.9 ± 1.2
Baseline neutrophil-to-lymphocyte ratio	2.9 ± 0.9	2.8 ± 0.8	2.9 ± 0.9
Baseline platelet-to-lymphocyte ratio	145 ± 40	148 ± 42	142 ± 38
Baseline SII	560 ± 160	545 ± 150	555 ± 155

Data presented as mean ± SD or % (n).

a Gender was self-reported by the patients.

### Randomization and Blinding

Patients were randomized in a 1:1:1 allocation ratio to one of three study groups using a computer-generated random sequence created before study initiation. Randomization was performed with variable block sizes to ensure balanced group allocation throughout the enrollment period. Group assignments were placed in sequentially numbered, sealed, opaque envelopes, which were opened only after patient enrollment.

Allocation concealment was maintained throughout the study. Study solutions were prepared by an independent anesthesiologist not involved in patient management, outcome assessment, or data analysis. The study drugs were prepared in identical syringes with the same volume and appearance for all groups.

Patients, surgeons, anesthesiologists responsible for intraoperative and postoperative care, outcome assessors, and statisticians were blinded to group allocation. The anesthesiologist performing the ESPB was not involved in postoperative data collection or outcome assessment. Blinding was maintained until completion of data analysis.

### Anesthesia and Surgical Procedure

All patients underwent standardized general anesthesia in accordance with institutional protocol. Total intravenous anesthesia was administered using propofol and remifentanil delivered via target-controlled infusion (TCI). Propofol was administered using a TCI pump according to the Schnider pharmacokinetic model, with an initial effect-site concentration of 3.0 to 4.0 µg × mL^-1^, titrated to maintain a bispectral index value between 40 and 60. Remifentanil was administered using TCI based on the Minto model, with effect-site concentrations ranging from 2.0 to 6.0 ng × mL^-1^, adjusted according to surgical stimulation and hemodynamic response. No long-acting opioids were administered intraoperatively. Standard intraoperative monitoring was performed in all patients and included electrocardiography, noninvasive blood pressure monitoring, pulse oximetry, capnography, and bispectral index monitoring. Anesthetic management, fluid therapy, and perioperative hemodynamic targets were standardized across all study groups.

Surgical procedures consisted of posterior lumbar decompression followed by multilevel instrumented fusion with pedicle screw fixation, performed by experienced spine surgeons using a standardized surgical technique. No additional intraoperative regional anesthesia techniques other than the study-related ESPB were used.

### ESPB Technique

Ultrasound-guided bilateral ESPB was performed at the L4 vertebral level after induction of general anesthesia and before surgical incision. A low-frequency curved ultrasound transducer was used to identify the transverse process and the overlying erector spinae muscle. After negative aspiration, the study solution was injected into the fascial plane deep to the erector spinae muscle, with the injection directed in a cranial (cephalad) direction.

Patients in the control group received 20 mL of 0.2% ropivacaine, combined with 2 mL of normal saline, on each side. In the dexamethasone group, 20 mL of 0.2% ropivacaine were combined with 4 mg of dexamethasone per side. In the dexmedetomidine group, 20 mL of 0.2% ropivacaine were combined with 50 µg of dexmedetomidine per side. The adjuvant doses were selected based on previously published clinical studies reporting effective, well-tolerated perineural use in regional anesthesia. The total injectate volume was identical across groups (40 mL), and the block technique was standardized.

### Postoperative Analgesia

Postoperative analgesia followed a standardized multimodal regimen applied uniformly in all study groups. As part of the nonopioid analgesic protocol, all patients received 1 g of intravenous acetaminophen every 6 hours and 1 g of intravenous metamizole every 8 hours during the postoperative period. NSAIDs, including ketoprofen and ibuprofen, were not administered perioperatively to avoid pharmacologic interference with postoperative inflammatory biomarker assessment.

Rescue analgesia consisted of intravenous morphine administered on patient request or when the Numeric Rating Scale (NRS) score exceeded 4. Morphine was given in 2-mg intravenous boluses, repeated as needed according to institutional protocol. Total opioid consumption during the first 48 postoperative hours was recorded and converted to morphine milligram equivalents (MME).

### Outcome Measures

The primary outcome was the time to first rescue opioid administration, defined as the time from completion of surgery to the first postoperative opioid dose, assessed within the first 48 postoperative hours.

The secondary outcome was total opioid consumption during the first 48 postoperative hours, expressed as MME. Postoperative pain intensity was assessed using the NRS (range 0 to 10) at 4, 8, 12, 16, 20, and 24 hours after surgery. Additional secondary outcomes included assessment of postoperative inflammatory response, as measured by the neutrophil-to-lymphocyte ratio and platelet-to-lymphocyte ratio, at predefined postoperative time points. The SII was calculated as the ratio of neutrophil and platelet counts to lymphocyte count. Changes in SII from baseline (ΔSII) were calculated as the difference between postoperative and preoperative SII values.

### Sample Size Calculation

Based on an institutional pilot study conducted at Poznań University of Medical Sciences, which included 24 patients (8 per group), a clinically relevant difference of 30% in time to first rescue opioid administration between groups was assumed. Patients included in the pilot study were not enrolled in the present randomized trial. With a two-sided alpha level of 0.05 and a statistical power of 80%, a minimum of 27 patients per group was required to detect a significant difference among the three groups. To account for potential dropouts or protocol deviations, the sample size was increased to 30 patients per group, resulting in a total planned enrollment of 90 patients.

### Exploratory Analyses

Exploratory analyses were performed to investigate potential associations between postoperative inflammatory response and analgesic requirements. Given its integrative nature, ΔSII was selected as the primary exploratory inflammatory marker. Within-group correlation analyses between ΔSII and cumulative opioid consumption were conducted to minimize the influence of between-group treatment effects and to explore potential mechanistic relationships related to sustained postoperative inflammation and rebound pain. Correlation analyses were considered exploratory and were not prespecified as primary or secondary outcomes; therefore, their results should be interpreted cautiously and viewed as generating hypotheses for future studies.

### Ethical Approval

The study protocol was approved by the bioethics committee at Poznań University of Medical Sciences (approval No. 545/23). The study was registered in ClinicalTrials.gov (NCT06233617) before patient enrollment. The trial was conducted in accordance with the Declaration of Helsinki and followed Consolidated Standards of Reporting Trials (CONSORT) guidelines. Written informed consent was obtained from all participants before inclusion.

### Statistical Analysis

Continuous variables are presented as mean ± SD and categorical variables are presented as percentages (numerators). Between-group comparisons were performed using ANOVA or the Kruskal-Wallis test, as appropriate. Pairwise comparisons were performed using the Tukey honestly significant difference test. Correlation analyses were performed using Spearman rho (ρ) because of the nonnormal distribution of selected variables. All statistical tests were two-sided, and p values < 0.05 were considered statistically significant. Statistical analyses were performed using standard statistical software. No adjustment for multiple comparisons was performed because secondary and exploratory analyses were not prespecified and were intended to generate hypotheses for future studies.

## Results

### Time to Opioid Rescue Dose

Patients receiving dexmedetomidine or dexamethasone had a longer time to first opioid administration than those receiving ropivacaine alone. Patients in the ropivacaine plus dexamethasone and ropivacaine plus dexmedetomidine groups experienced a longer time to first opioid administration compared with the control group. The time to first opioid administration was 6.2 ± 0.8 hours with ropivacaine alone versus 12.7 ± 2.7 hours with dexamethasone (mean difference 6.6 hours [95% confidence interval (CI) 5.6 to 7.7]; p < 0.001) and 15.5 ± 2.0 hours with dexmedetomidine (mean difference 9.6 hours [95% CI 8.5 to 10.5]; p < 0.001). Dexmedetomidine also prolonged time to opioid administration compared with dexamethasone (mean difference 2.8 hours [95% CI 1.80 to 3.84]; p < 0.001) (Table 2).

**Table 2.** Postoperative pain outcomes and opioid consumption

Variable	Ropivacaine alone (R) (n = 30)	Ropivacaine with dexamethasone (DEX) (n = 30)	Ropivacaine with dexmedetomidine (DEM) (n = 30)	Mean difference (95% CI)	p value
Time to first rescue analgesia in hours	6.2 ± 0.8	12.7 ± 2.7	15.5 ± 2.0		
R vs DEX				6.6 (5.6 to 7.7)	< 0.001
R vs DEM				9.6 (8.5 to 10.5)	< 0.001
DEX vs DEM				2.8 (1.8 to 3.8)	< 0.001
Total opioid consumption in MMEq	57.9 ± 7.9	29.4 ± 4.6	35.1 ± 5.1		
R vs DEX				28.5 (24.7 to 32.8)	< 0.001
R vs DEM				22.8 (19.0 to 26.6)	< 0.001
DEX vs DEM				5.7 (1.9 to 9.5)	0.02
NRS^a^					
4 hours	6.1 ± 0.7	3.2 ± 1.0	3.9 ± 0.7		
R vs DEX				2.9 (2.4 to 3.4)	< 0.001
R vs DEM				2.2 (1.8 to 2.7)	< 0.001
DEX vs DEM				-0.7 (-1.2 to -0.1)	0.01
8 hours	5.7 ± 0.7	3.3 ± 0.7	2.8 ± 0.6		
R vs DEX				2.3 (1.9 to 2.8)	< 0.001
R vs DEM				2.9 (2.5 to 3.3)	< 0.001
DEX vs DEM				0.6 (0.2 to 1.0)	0.005
12 hours	5.7 ± 0.5	3.7 ± 0.5	2.3 ± 0.5		
R vs DEX				2.0 (1.7 to 2.3)	< 0.001
R vs DEM				3.3 (3.0 to 3.7)	< 0.001
DEX vs DEM				1.3 (1.0 to 1.7)	< 0.001
16 hours	4.9 ± 0.6	2.7 ± 0.5	3.6 ± 0.5		
R vs DEX				2.2 (1.9 to 2.5)	< 0.001
R vs DEM				1.3 (0.9 to 1.6)	< 0.001
DEX vs DEM				-0.9 (-1.2 to -0.6)	< 0.001
20 hours	4.4 ± 0.9	2.9 ± 0.6	3.7 ± 0.7		
R vs DEX				1.5 (1.0 to 1.9)	< 0.001
R vs DEM				0.5 (0.1 to 1.0)	0.03
DEX vs DEM				-0.9 (-1.3 to -0.5)	< 0.001
24 hours	4.4 ± 0.6	2.8 ± 0.8	3.3 ± 0.5		
R vs DEX				1.6 (1.1 to 2.0)	< 0.001
R vs DEM				1.0 (0.7 to 1.4)	< 0.001
DEX vs DEM				-0.5 (-0.9 to -0.1)	< 0.01

Data presented as mean ± SD. Mean differences are expressed as comparator minus control with corresponding 95% CI. p values refer to between-group comparisons at each time point.

a 0 = no pain, 10 = worst pain imaginable.

Adding ropivacaine plus dexamethasone adjuvant to the ESPB resulted in longer time to first opioid rescue dose for NRS scores > 4 (control 6.2 ± 0.8 hours versus ropivacaine plus dexamethasone 2.7 ± 2.7 hours, mean difference 6.6 hours [95% CI 5.63 to 7.67]; p < 0.001); and likewise, adding ropivacaine plus dexmedetomidine resulted in longer time to first opioid rescue dose (Table 2).

### Total Opioid Consumption and Pain Scores

Patients receiving dexamethasone or dexmedetomidine used less opioid during the first 48 postoperative hours than those receiving ropivacaine alone. Total opioid consumption was 29.4 ± 4.6 MME for dexamethasone and 35.1 ± 5.1 MME for dexmedetomidine compared with 57.9 ± 7.9 MME for ropivacaine alone. Pain scores were lower in both adjuvant groups than in the ropivacaine alone group, with differences of approximately 2 to 3 points at early time points (4 to 8 hours) and 1 to 2 points at later time points. The early differences exceeded the minimum clinically important difference for postoperative pain, whereas later differences were smaller.

### Inflammatory Markers

Postoperative inflammatory response was lowest in patients receiving dexamethasone, intermediate with dexmedetomidine, and highest with ropivacaine alone, as reflected by changes in SII (ΔSII). The increase in ΔSII at 48 hours was approximately + 821 with ropivacaine alone, + 394 with dexmedetomidine, and + 184 with dexamethasone (Table 3). Similar patterns were observed for neutrophil-to-lymphocyte ratio, whereas differences in platelet-to-lymphocyte ratio were smaller and more variable over time.

**Table 3.** Postoperative inflammatory biomarkers and systemic immune response

Value	Ropivacaine alone (R) (n = 30)	Ropivacaine with dexamethasone (DEX) (n = 30)	Ropivacaine with dexmedetomidine (DEM) (n = 30)	Mean difference (95% CI)	p value
Neutrophil-to-lymphocyte ratio					
12 hours	6.07 ± 1.5	4.07 ± 0.7	5.2 ± 0.7		
R vs DEX				2.0 (1.4 to 2.6)	< 0.001
R vs DEM				0.9 (0.3 to 1.6)	< 0.001
DEX vs DEM				-1.1 (-1.7 to -0.5)	< 0.001
24 hours	6.1 ± 1.0	4.9 ± 0.9	6.0 ± 0.9		
R vs DEX				1.3 (0.7 to 1.8)	< 0.001
R vs DEM				0.2 (-0.4 to 0.7)	0.74
DEX vs DEM				-1.1 (-1.7 to 0.5)	< 0.001
48 hours	7.1 ± 1.0	4.2 ± 1.0	5.5 ± 1.0		
R vs DEX				2.8 (2.4 to 3.5)	< 0.001
R vs DEM				1.5 (0.9 to 2.1)	< 0.001
DEX vs DEM				-1.3 (-1.9 to -0.7)	< 0.001
Platelet-to-lymphocyte ratio					
12 hours	172 ± 43	162 ± 37	167 ± 38		
R vs DEX				10 (-10.5 to 30.5)	0.33
R vs DEM				5 (-15.3 to 25.3)	0.62
DEX vs DEM				-5 (-24.8 to 14.8)	0.61
24 hours	202 ± 47	172 ± 38	187 ± 42		
R vs DEX				30 (8.1 to 51.9)	< 0.01
R vs DEM				15 (-6.5 to 36.5)	0.17
DEX vs DEM				-15 (-36.2 to 6.2)	0.16
48 hours	221 ± 52	167 ± 35	185 ± 41		
R vs DEX				54 (32.4 to 75.6)	< 0.001
R vs DEM				36 (13.8 to 58.2)	< 0.01
DEX vs DEM				-18 (-37.9 to 1.9)	0.08
SII					
12 hours	934 ± 253	784 ± 231	853 ± 321		
R vs DEX				150 (22 to 278)	< 0.05
R vs DEM				81 (-66 to 228)	0.27
DEX vs DEM				-69 (-212 to 74)	0.34
24 hours	1253 ± 329	847 ± 234	1004 ± 267		
R vs DEX				406 (262 to 550)	< 0.001
R vs DEM				249 (95 to 403)	< 0.01
DEX vs DEM				-157 (-287 to -27)	< 0.05
48 hours	1358 ± 332	751 ± 214	954 ± 271		
R vs DEX				607 (473 to 741)	< 0.001
R vs DEM				404 (252 to 556)	< 0.001
DEX vs DEM				-203 (-324 to -82)	0.001
ΔSII					
12 hours	+ 354	+ 248	+ 297		
R vs DEX				106 (58 to 154)	< 0.001
R vs DEM				57 (9 to 105)	< 0.05
DEX vs DEM				-49 (-97 to -1)	< 0.05
24 hours	+ 703	+ 287	+ 456		
R vs DEX				416 (342 to 490)	< 0.001
R vs DEM				247 (173 to 321)	< 0.001
DEX vs DEM				-169 (-243 to -95)	< 0.001
48 hours	+ 821	+ 184	+ 394		
R vs DEX				637 (563 to 711)	< 0.001
R vs DEM				427 (353 to 501)	< 0.001
DEX vs DEM				-210 (-284 to -136)	< 0.001

Data presented as mean ± SD. Mean differences are expressed as control minus comparator with corresponding 95% CIs. SII = neutrophils × platelets / lymphocytes. ΔSII = change in SII from baseline. p values refer to pairwise between-group comparisons at each postoperative time point.

SII increased in all groups, but the magnitude of increase was smallest with dexamethasone and greatest with ropivacaine alone. The ropivacaine plus dexamethasone group demonstrated the lowest postoperative SII values and the smallest ΔSII at 24 and 48 hours, followed by the ropivacaine plus dexmedetomidine group, whereas the control group exhibited the greatest increase. Between-group differences in ΔSII were statistically significant at all assessed time points (all p < 0.0001).

Exploratory within-group correlation analyses were performed to assess the relationship between ΔSII at 48 hours and cumulative opioid consumption during the first 48 postoperative hours. No meaningful correlations were observed between ΔSII and opioid consumption within groups (Fig. 2). Correlation coefficients have been provided (Supplemental Table 1; http://links.lww.com/CORR/B547).

**Fig. 2:**
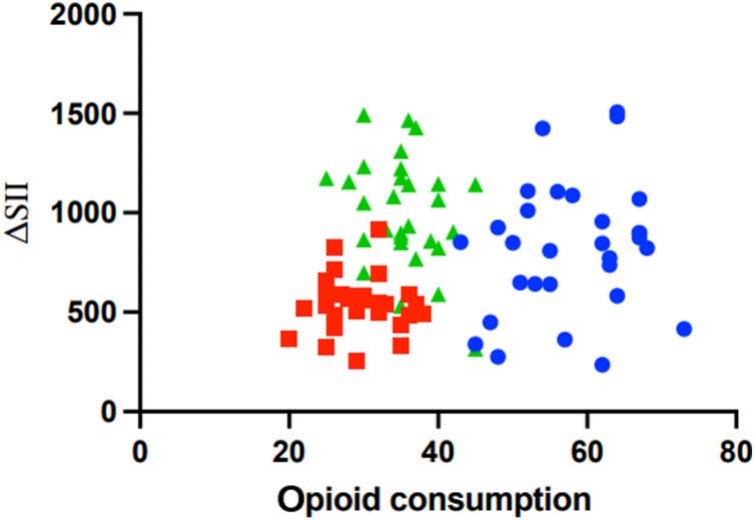
Within-group correlations between changes in SII (ΔSII) at 48 hours postoperatively and cumulative opioid consumption during the first 48 postoperative hours. Scatter plots are shown separately for the ropivacaine alone (blue circles), ropivacaine with dexamethasone (red squares), and ropivacaine with dexmedetomidine (green triangles) groups. Spearman correlation coefficients (ρ) are provided for each group. No statistically significant correlations were observed, indicating that between-group differences in opioid consumption and inflammatory response were not driven by linear patient-level associations. Control group ρ = 0.17; ropivacaine with dexamethasone group ρ = -0.07; ropivacaine plus dexmedetomidine group ρ = -0.18. A color image accompanies the online version of this article.

## Discussion

This study addressed an important clinical problem, as postoperative pain after lumbar spine surgery is closely linked to opioid use and systemic inflammation. Although perineural adjuvants are widely used with ESPB, their comparative effects on pain dynamics and inflammatory response remain unclear. We found that dexmedetomidine primarily prolongs early postoperative analgesia, whereas dexamethasone reduces overall opioid use and attenuates the postoperative inflammatory response. These findings suggest that clinicians can tailor adjuvant selection based on the desired analgesic profile, balancing early pain control against longer term recovery and inflammation [17, 28].

### Limitations

Several limitations should be acknowledged. First, this was a single-center study, which may limit generalizability. Second, although patients with opioid abuse were excluded, detailed quantification of prior intermittent opioid exposure was not systematically captured, and residual confounding related to preoperative analgesic use cannot be excluded. Third, the study population was relatively homogeneous with respect to surgical setting and perioperative care, but we had limited ability to account for broader clinical variation in pain medication requirements related to age, comorbidities, pain sensitivity, and underlying degenerative conditions. Fourth, the inflammatory markers assessed were indirect measures of systemic inflammation and do not capture local tissue-level immune responses [5, 13, 25]. Fifth, the exploratory correlation analyses were hypothesis generating and not powered for mechanistic patient-level associations [14, 20]. Finally, long-term pain outcomes, functional recovery, hospital length of stay, and rehabilitation-related endpoints were not predefined outcomes and should be addressed in future multicenter studies [8, 29]. In addition, the trial did not include either a no-ESPB comparator or an intravenous adjuvant comparator; therefore, the present results should be interpreted as a comparison between perineural adjuvant strategies within a standardized ESPB-based analgesic pathway rather than as a study of ESPB efficacy itself or route-specific superiority [11, 21, 26, 29].

### Time to Opioid Rescue Dose

Dexmedetomidine was associated with a longer time to first opioid administration, indicating superior early postoperative analgesia. This finding suggests a predominantly early analgesic effect, likely related to modulation of peripheral nociceptive transmission and enhanced block characteristics [1, 10]. Prior studies have shown that dexmedetomidine prolongs block duration and improves early pain control, which supports our observations [21]. These findings may be clinically relevant when immediate postoperative analgesia is the primary goal, particularly in patients with high early pain intensity.

### Total Opioid Consumption and Pain Scores

In contrast, dexamethasone was associated with lower cumulative opioid consumption over 48 hours and lower pain scores over time. This suggests a more sustained analgesic effect, likely related to attenuation of postoperative inflammation and modulation of delayed nociceptive input [14, 20]. Previous studies and meta-analyses have demonstrated that perineural dexamethasone prolongs analgesia and reduces opioid requirements, often exceeding the effect of intravenous administration, suggesting a local mechanism of action [8, 14, 20]. Clinically, this may reduce prolonged opioid exposure and the risk of rebound pain after block resolution [3, 30].

### Inflammatory Markers

Dexamethasone was associated with the lowest postoperative inflammatory response, as reflected by lower SII and ΔSII values. SII has been increasingly recognized as a robust marker of surgical stress and systemic inflammation, integrating neutrophils, platelets, and lymphocytes [2, 5, 7, 27]. These findings support the concept that postoperative pain is closely linked to systemic inflammation. By attenuating the inflammatory cascade, dexamethasone may reduce delayed nociceptive input and opioid demand [15, 25]. In contrast, dexmedetomidine had a more limited effect on inflammatory markers, which may explain the differences in later pain trajectories. These findings suggest that antiinflammatory modulation may play an important role in improving postoperative recovery beyond immediate analgesia.

These results indicate that dexmedetomidine and dexamethasone exert distinct and complementary effects on postoperative recovery. Dexmedetomidine primarily enhances early nociceptive modulation, whereas dexamethasone provides sustained antiinflammatory and antirebound effects [28]. This distinction is clinically relevant, as it suggests that analgesic efficacy alone does not fully capture the biological impact of perineural adjuvants in major spine surgery.

### Conclusion

In summary, perineural dexamethasone and dexmedetomidine act as distinct modulators of postoperative pain and inflammation when used as adjuvants to ESPB in lumbar spine surgery. Dexmedetomidine provides superior early analgesia, whereas dexamethasone more effectively attenuates postoperative inflammatory response and reduces cumulative opioid consumption. These findings support a mechanism-informed approach to selecting perineural adjuvants based on the desired temporal analgesic profile. Dexamethasone appears to provide more sustained antiinflammatory and opioid-sparing effects, particularly in patients at higher risk. This combination may be especially relevant within enhanced recovery pathways for spine surgery.
